# Predictors of Critical Acute Pancreatitis: A Prospective Cohort Study

**DOI:** 10.1097/MD.0000000000000108

**Published:** 2014-10-31

**Authors:** Lu Ke, Zhi-hui Tong, Wei-qin Li, Congye Wu, Ning Li, John A. Windsor, Jie-shou Li, Maxim S. Petrov

**Affiliations:** Department of General Surgery (LK, ZT, WL, CW, NL, JL), Jinling Hospital, Nanjing University School of Medicine, Nanjing, China; and Department of Surgery (JAW, MSP), University of Auckland, Auckland, New Zealand.

## Abstract

Critical acute pancreatitis (CAP) has recently emerged as the most ominous severity category of acute pancreatitis (AP). As such there have been no studies specifically designed to evaluate predictors of CAP. In this study, we aimed to evaluate the accuracy of 4 parameters (Acute Physiology and Chronic Health Evaluation [APACHE] II score, C-reactive protein [CRP], D-dimer, and intra-abdominal pressure [IAP]) for predicting CAP early after hospital admission. During the study period, data on patients with AP were prospectively collected and D-dimer, CRP, and IAP levels were measured using standard methods at admission whereas the APACHE II score was calculated within 24 hours of hospital admission. The receiver-operating characteristic (ROC) curve analysis was applied and the likelihood ratios were calculated to evaluate the predictive accuracy. A total of 173 consecutive patients were included in the analysis and 47 (27%) of them developed CAP. The overall hospital mortality was 11% (19 of 173). APACHE II score ≥11 and IAP ≥13 mm Hg showed significantly better overall predictive accuracy than D-dimer and CRP (area under the ROC curve—0.94 and 0.92 vs 0.815 and 0.667, correspondingly). The positive likelihood ratio of APACHE II score is excellent (9.9) but of IAP is moderate (4.2). The latter can be improved by adding CRP (5.8). In conclusion, of the parameters studied, APACHE II score and IAP are the best available predictors of CAP within 24 hours of hospital admission. Given that APACHE II score is rather cumbersome, the combination of IAP and CRP appears to be the most practical way to predict critical course of AP early after hospital admission.

## INTRODUCTION

The clinical course of acute pancreatitis (AP) greatly varies between patients and this makes the accurate classification and prediction of disease severity very important for both clinical decision-making and research recruitment. In 1992, the Atlanta Symposium provided an international consensus on the severity classification of AP (mild and severe) and the definitions of a number of systemic and local complications (including “organ failure [OF],” “pancreatic necrosis,” “acute fluid collection,” and “pancreatic abscess”).^1^ Over the past 20 years, with better understanding of pathophysiology of AP and its complications, improved diagnostic imaging, and the recognition of different subgroups of patients with different clinical courses and outcomes, there was recognition that the binary severity classification of AP was inadequate.

Recently, the determinant-based classification (DBC) of AP severity was systematically introduced to classify AP severity into 4 categories (mild, moderate, severe, and critical) based on the presence or absence of local and systemic determinants and their interaction.^2^ A particular strength of the new classification is identification of a subgroup of patients with the combination of persistent OF and infected pancreatic necrosis, an overwhelming mortality, which has been defined as “critical” acute pancreatitis (CAP).^3,4^ Prospective validation of this subgroup has been published.^5–7^ However, it is not known whether it is possible to accurately predict the development of CAP, especially early in the course of disease. A reliable tool for accurate prediction of CAP is essential for the institution of measures to reduce eventual severity and mortality and to enable the accurate enrollment of patients into clinical studies.

During the past decades, a wide array of predictive factors and scoring systems have been introduced and evaluated for the identification of patients who are at high risk of developing severe AP (as defined by the original Atlanta classification) and dying.^8,9^ In this study, we aimed to evaluate the accuracy of 2 more frequently used (Acute Physiology and Chronic Health Evaluation [APACHE] II score and C-reactive protein [CRP]) and 2 less frequently used (D-dimer and intra-abdominal pressure [IAP]) parameters for predicting CAP. All 4 predictors have been shown to be of value in predicting severe AP (as defined by the original Atlanta classification),^8,10–13^ but there have not been any studies evaluating these factors (alone and in combination) in predicting CAP.

## METHODS

### Patients

**FIGURE 1:**
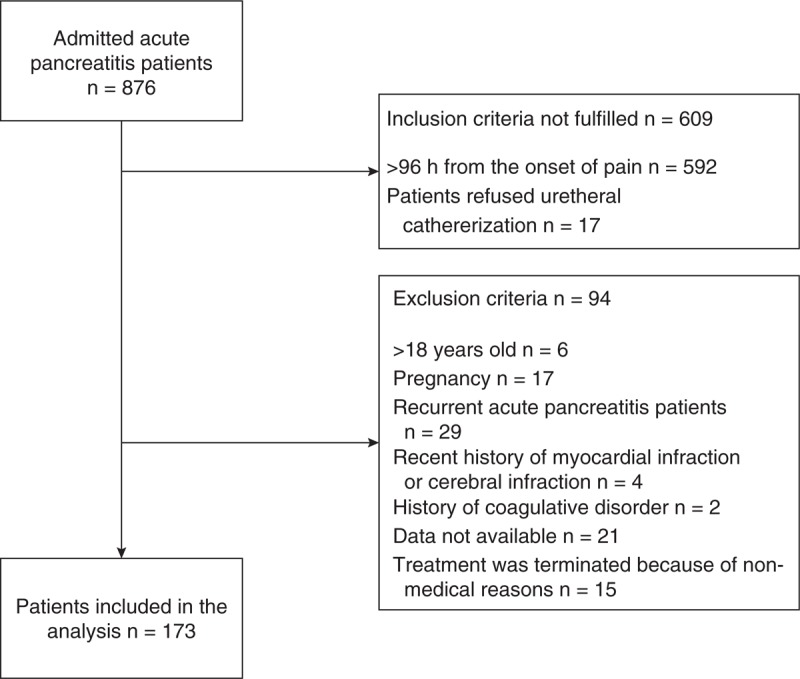
Flow chart of the study.

All patients admitted to Jinling Hospital (Nanjing, China), a 2000-bed tertiary referral center, with a diagnosis of AP between January 2009 and March 2013 were considered for enrollment. The study inclusion criteria were diagnosis of AP and admission to Jinling Hospital within 96 hours after onset of symptoms. Patients were excluded if they were <18 years, they were pregnant, they had suffered previous attacks of AP, they had a known history of coagulative disorders or a recent history of myocardial infarction or cerebral infarction, they had developed CAP, data on studied parameters (IAP, D-dimer, CRP on admission, APACHE II score within first 24 hours) were not available, and treatment was terminated because of nonmedical reasons. All the patients initially received standard conservative treatment according to the recent international guidelines.^14,15^ In our center, urethral catheter was routinely placed for measuring both hourly urine output and IAP. OF was treated with organ-specific support if needed, including mechanical ventilation, continuous renal replacement therapy, vasoactive agents, and others. Infected (peri)pancreatic necrosis (IPN) was managed with step-up approach including percutaneous or endoscopic drainage as the first-line approach. The patients underwent surgical necrosectomy when the drainage failed. Study flow chart and reasons for exclusion could be seen in Figure 1. Ethical approval was waived in our institute based on the observational nature of this study and written informed consent was obtained from all patients or their representative for publication of data.

### Definitions

Diagnosis of AP was based on abdominal pain suggestive of AP, serum amylase at least 3 times the upper limit of normal, and/or characteristic findings of AP on computed tomography.^1^ CAP was defined as the presence of both persistent OF and IPN.^2^ The criteria for OF were described for 3 organ systems: cardiovascular (need for inotropic agent), renal (creatinine ≥171 µmol/L), and respiratory (PaO_2_/FIO_2_ ≤ 300 mm Hg). Persistent OF was defined as OF in the same organ system for 48 hours or more. IPN was confirmed when 1 or more of the following were present: gas bubbles within (peri)pancreatic necrosis on computed tomography; a positive culture of (peri)pancreatic necrosis obtained by image-guided fine-needle aspiration; a positive culture of (peri)pancreatic necrosis obtained during the first drainage and/or necrosectomy. The category of severity for each patient was confirmed after discharge or hospital death.

### Data Collection

In all patients enrolled in this study, blood samples were obtained on admission for the measurement of plasma D-dimer and CRP levels. The samples were processed by the Central Laboratory of Jinling Hospital using the standard methods. The normal ranges for D-dimer and CRP were 0 to 0.5 mg/L and 0 to 8 mg/L, respectively. IAP was determined at the same time with a catheter inserted into the bladder according to the standard technique recommended by the World Society of Abdominal Compartment Syndrome.^16^ The normal range of IAP is 5 to 7 mm Hg in critically ill adults and intra-abdominal hypertension (IAH) is defined by a sustained or repeated pathological elevation in IAP ≥12 mm Hg.^16^ APACHE II score was calculated on the basis of the worst values during the first 24 hours after admission. Baseline data and clinical outcome variables were also recorded. All patients were followed until discharge from the hospital or hospital mortality.

### Statistical Analysis

Continuous variables were expressed as mean with standard deviations and categorical variables were described in absolute numbers and percentages. The Shapiro–Wilk test was used to confirm normal distribution of the values prior to analysis. Between-group analysis was performed by analysis of variance for factorial analysis or Kruskal–Wallis test. The significance of differences in proportions was tested by χ^2^ test. The prognostic performances of the studied predictors were further assessed by calculating sensitivity, specificity, positive predictive value (PPV), negative predictive value (NPV), positive likelihood ratio (PLR), and negative likelihood ratio (NLR). The post-test probability of CAP was derived for each predictor from the likelihood ratio nomogram and compared with the pre-test probability of CAP (reference). The discriminative ability of each predictor studied was also evaluated by calculating respective areas under the curve (AUC) using receiver-operating characteristic (ROC) curves. The AUC ranges were, by convention, between 1.0 (perfect separation of the 2 groups by the test) and 0.5 (no ability of the test to distinguish between the 2 groups). The optimal cutoffs were the values yielding maximum sums of sensitivity and specificity from the ROC curves.^17,18^ The Z statistic was used for pairwise comparison of ROC curves. Stepwise logistic regression was then used to determine the accuracy of different combinations of the 3 single predictors (CRP, D-dimer, and IAP) in predicting the critical course of AP on admission.^19^ APACHE II score was not included in the combined analysis as it is a multifactor scoring system itself. Moreover, to evaluate the relative improvement of the DBC in discriminating AP severity, we calculated a net reclassification improvement (NRI) using the methods previously described.^20^ All statistical tests were 2-tailed, and the significance level was set at *P* < 0.05. Data were analyzed using SPSS 17.0 for Windows (SPSS, Chicago, IL).

## RESULTS

### Overall Patient Characteristics and Outcomes

A total of 173 eligible patients were included: 59 (34%) of them had mild AP, 41 (24%) had moderate AP, 26 (15%) had severe AP, and 47 (27%) had CAP. The demographic characteristics of the 4 categories were similar (Table 1). The overall hospital mortality was 11% (19 of 173). With regard to local determinants of severity, (peri)pancreatic necrosis developed in 105 (61%) patients, and nearly a half of them had pancreatic infection (49%, 51 of 105). Only 17 (33%) patients with IPN underwent surgical intervention and mortality in this subgroup was high (59%, 10 of 17). Percutaneous and/or endoscopic drainage only was used in most patients with IPN (67%, 34 of 51) and most of them survived (74%, 25 of 34). With regard to systemic determinants of severity, OF developed in 89 (51%) patients and the majority of them had persistent OF (78%, 69 of 89). OF of 2 systems was diagnosed in 23 patients and 20 patients developed OF in all the 3 organ systems. The median time from onset of AP to establishing the diagnosis of CAP was 20 days (interquartile range, 16–28 days).

**TABLE 1:**
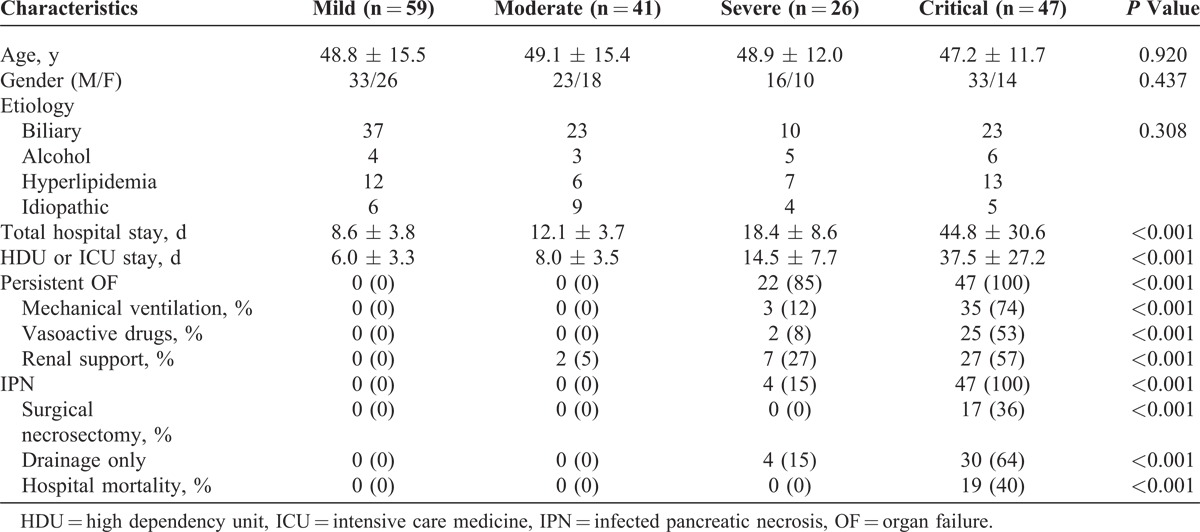
Demographic Characteristics and Clinical Outcomes of Patients With Acute Pancreatitis With Different Categories of Severity

### Comparing Management and Outcomes for Different Categories of Severity

**FIGURE 2:**
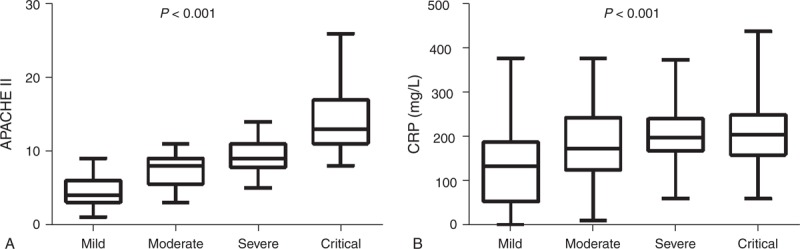
Levels of APACHE II and CRP score on admission in patients with different severities of acute pancreatitis. CRP = C-reactive protein.

The values of APACHE II score and CRP on admission in the 4 groups are presented in Figure 2. The 4 categories of severity differed significantly (*P* < 0.001) in terms of mortality, total length of hospitalization, and length of intensive care unit (ICU) stay (Table 1). The need for organ support and interventions also significantly differed between the 4 categories (Table 1). Moreover, the DBC showed excellent discriminative ability over the original Atlanta classification as evidenced by an NRI of 108.4% (Table 2).

**TABLE 2:**
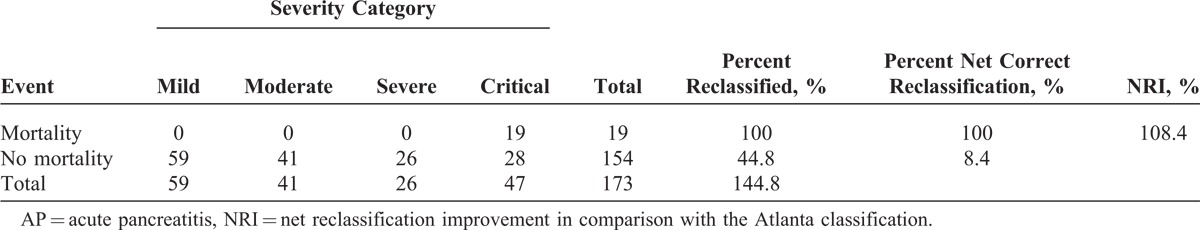
NRI for Mortality in AP With the Use of the Determinant-Based Classification of AP Severity

### Individual Predictors of CAP

**FIGURE 3:**
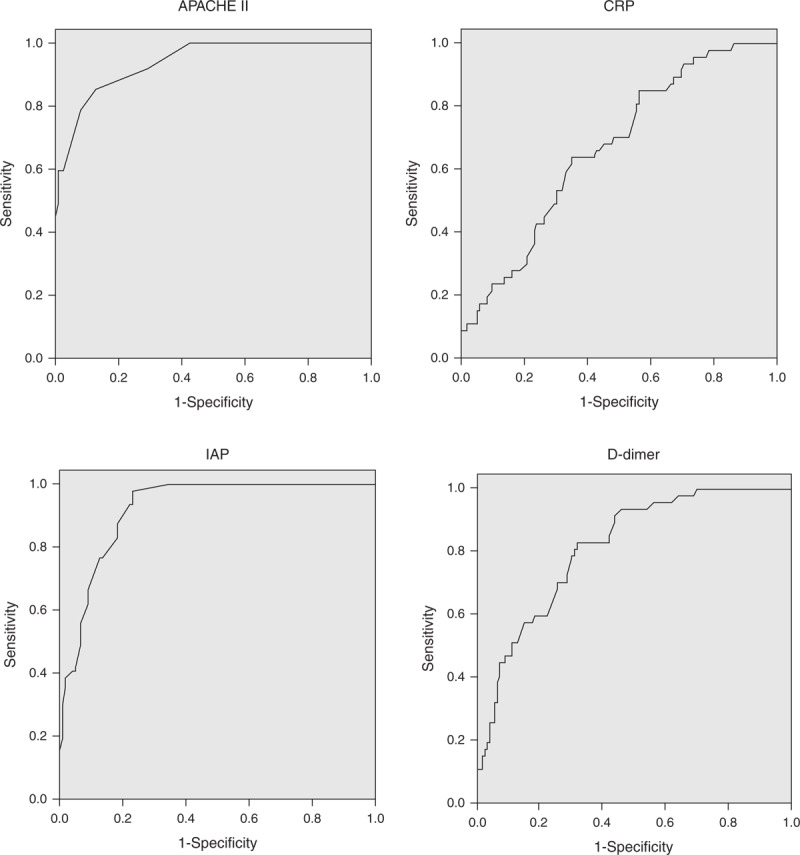
ROC curves for APACHE II score, CRP, IAP, and D-dimer in predicting the development of critical acute pancreatitis. CRP = C-reactive protein, IAP = intra-abdominal pressure, ROC = receiver-operating characteristic.

ROC curves were drawn to evaluate the accuracy of APACHE II score, CRP, D-dimer, and IAP in predicting the CAP (Figure 3). The AUC values for APACHE II score and IAP were significantly higher than for CRP and D-dimer (*P* < 0.05) but there was no statistically significant difference between APACHE II score and IAP (*P* > 0.05). Sensitivity, specificity, PPV, NPV, PLR, and NLR for all the studied parameters are presented in Table 3. Optimal cutoff points for each predictor were derived from the ROC curves. Both CAP and mortality were observed more frequently in patients with CRP, D-dimer, IAP, and APACHE II score levels above the optimal cutoff values (Table 4).

**TABLE 3:**
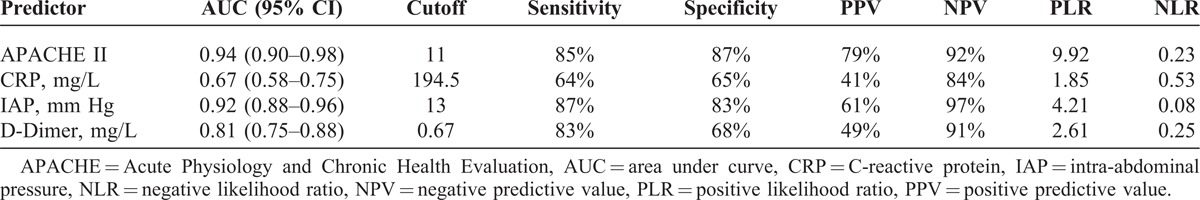
Accuracy of the Studied Parameters in Predicting Critical Acute Pancreatitis

**TABLE 4:**
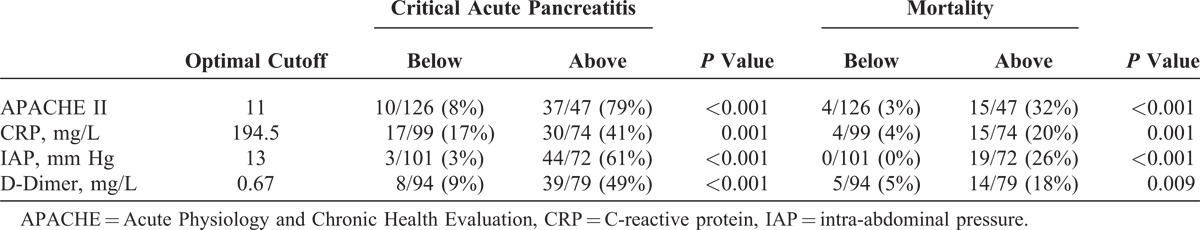
Incidence Rate of Critical Acute Pancreatitis and Mortality in Patients With the Predictor’s Levels Above and Below Optimal Cutoffs

The logistic regression analysis demonstrated that every 1 mm Hg increase of IAP above the optimal cutoff was associated with more than a 50% increase in mortality (odds ratio [OR] 1.56; 95% confidence interval [CI] 1.33–1.84; *P* < 0.001). Every 0.1 mg/L increase in concentration of D-dimer above the optimal cutoff was associated with more than a 10% increase in mortality (OR 1.12; 95% CI 1.03–1.22; *P* = 0.01). Every 10 mg/L increase in concentration of CRP above the optimal cutoff was associated with approximately 4% increase in mortality (OR 1.04; 95% CI 0.983–1.102; *P* = 0.17).

### Combination of Predictors of CAP

**FIGURE 4:**
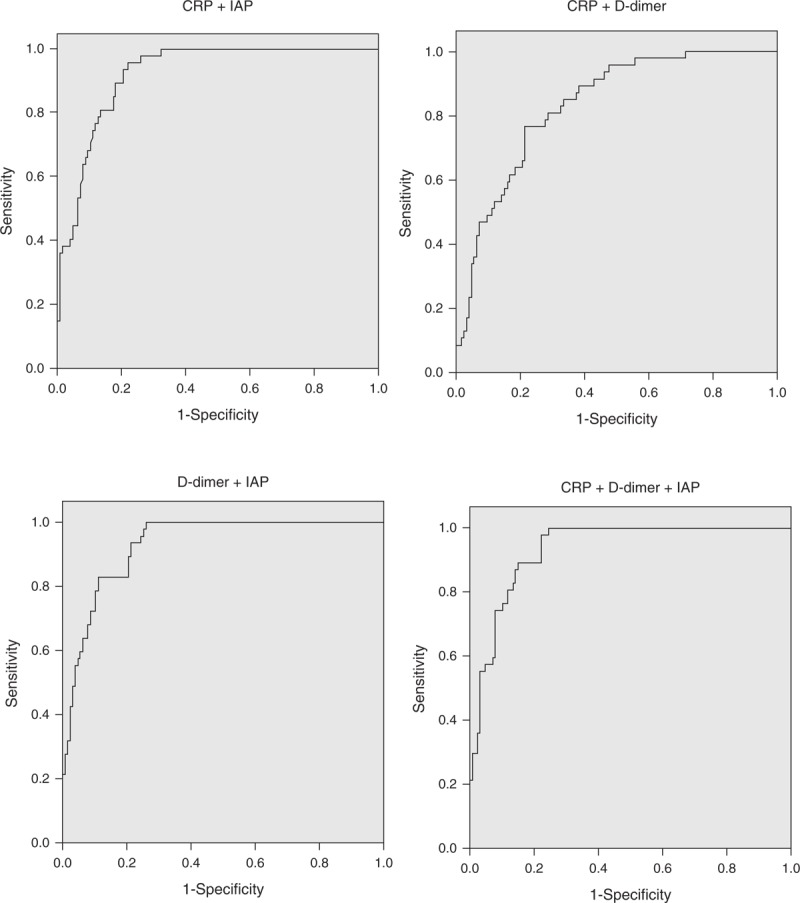
ROC curves for combinations of 3 single-factor parameters in predicting the development of critical acute pancreatitis. ROC = receiver-operating characteristic.

Combining D-dimer with CRP resulted in an increase of AUC from 0.67 (0.58 to 0.75) to 0.83 (0.77 to 0.90) and this difference was statistically significant (*P* < 0.05). Adding D-dimer to IAP resulted in an increase of AUC from 0.92 (0.88 to 0.96) to 0.93 (0.89 to 0.97) but this difference was not statistically significant (*P* > 0.05). Combining CRP and IAP did not result in an increase of AUC. Combining of CRP, D-dimer, and IAP resulted in an increase of AUC from 0.92 (0.88 to 0.96) to 0.93 (0.90 to 0.97) but this difference was not statistically significant (*P* > 0.05) (Figure 4). Sensitivity, specificity, PPV, NPV, PLR, and NLR for all the combination of predictors are presented in Table 5.

**TABLE 5:**
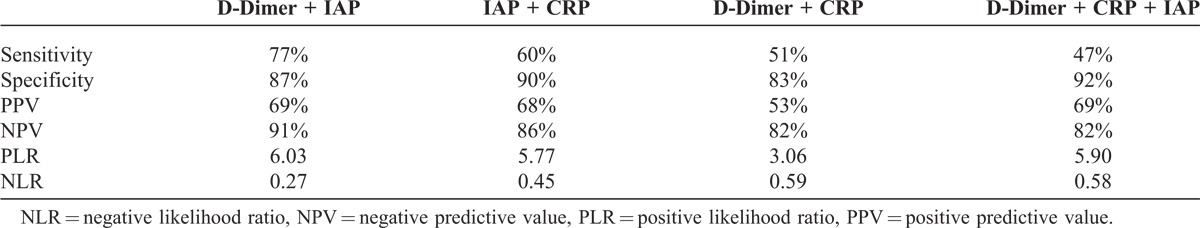
Predictive Values and Likelihood Ratios of Various Combinations of Predictors Based on the Optimal Cutoff Points

## DISCUSSION

Systemic and local complications determine mortality in patients with AP and this has been highlighted in DBC.^21–25^ Patients with CAP have the highest risk of mortality as they have both persistent OF and IPN. Therefore, accurate prediction of the development of CAP is an important clinical goal, which could help to initiate appropriate treatment earlier and reduce mortality. The key finding here is that APACHE II score and IAP could offer a very good accuracy in predicting CAP whereas the accuracy of D-dimer and CRP is not good enough to be used as a sole predictor of CAP.

To the best of our knowledge, this is the first study to investigate predictors of CAP. Three individual parameters (IAP, D-dimer, and CRP) and a multifactor scoring system (APACHE II score) were assessed in this pilot cohort study. The results demonstrate that APACHE II score ≥11 performed best for the prediction of the critical course as evidenced by AUC of 0.94 and PLR of nearly 10, which indicates moderate-to-large increase in the likelihood of mortality. The optimal threshold of APACHE II score for early identification of those patients who are at highest risk of death is 11, and hence, the cutoff of 8, advocated in the original Atlanta classification, may need to be abandoned.^1,26^ However, the major limitation of APACHE II score is its complexity and the ideal predictor of CAP would have to be an individual predictor or a combination of few relatively simple predictors.

Of the 3 individual predictors investigated, IAP offers the best diagnostic accuracy, and based on the logistic regression analysis, every 1 mm Hg increase of IAP above the optimal cutoff is associated with a 50% increase in mortality. In fact, its AUC of 0.92 is very close to that of APACHE II score but the PLR of 4.2 suggests only a moderate increase in the likelihood of mortality (Table 3). However, it is worth noting that patients with an IAP below the cutoff value (13 mm Hg) could almost certainly be ruled out for the development of CAP and mortality (Table 4). Increased IAP is a common finding in patients with AP and is reported to be strongly associated with unfavorable outcomes, especially when abdominal compartment syndrome (ACS) develops.^13,27–31^ The mechanisms underlying the development of IAH in AP include ascites and multifluid collections caused by inflammatory process sourced from pancreas, impaired gastrointestinal motility, severe edema, gastric dilation, ileus, and some iatrogenic factors, such as inappropriate fluid resuscitation.^27^ Once IAH/ACS occurs, it can initiate a vicious circle by itself, as visceral microcirculation in the pancreas is further compromised^32–35^ and as decreased cardiac output and elevated intrathoracic pressure further compromise the oxygen delivery.^36,37^ All of these could ultimately aggravate AP leading to the critical course. However, many factors causing IAH/ACS could be alleviated with adequate management, for example, fluid collections with timely percutaneous drainage. This may explain why the PLR of IAP is inferior to that of APACHE II score, which could reflect the underlying pathophysiology more comprehensively.

APACHE II score could serve as a good predictive tool for CAP, but it is a complex scoring system that includes 18 parameters and is of limited use in a routine management of patients with AP.^38^ Therefore, this study has also evaluated possible combinations of the 3 sole predictors, aiming to find a combination that is practical and reasonably reliable. In terms of accuracy, the best 2-factor combinations include IAP, and the predictive metrics were comparable between IAP + D-dimer (improved PLR from 4.2 to 6.0) and IAP + CRP (improved PLR from 4.2 to 5.8). Given that CRP is routinely used in patients with AP whereas D-dimer is not, IAP and CRP seem to be the combination of choice in early prediction of CAP. Combination of all the 3 sole parameters did not show a significantly improved accuracy when compared with the combinations above and thus the former is not advocated for clinical use.

In line with other recent studies that investigated the discriminative ability of DBC,^5–7^ this study has confirmed the clinical validity of the 4 categories, as evidenced by significant difference between all the 4 categories, for important clinical outcomes such as mortality, total hospital and ICU stay, need for drainage/necrosectomy, as well as need for mechanical ventilation, vasoactive agents, and renal support. However, all the 3 studies included very limited number of patients with CAP—3 (0.6% of total),^7^ 17 (6.6% of total),^5^ and 8 (5.3% of total).^6^ In contrast, CAP in the present study accounts for more than a quarter of all episodes (27.2%), likely because Jinling Hospital has a catchment area of approximately 80 million people and is also a nationwide transfer center for AP. Unlike other studies mentioned above, this study has also quantified the relative improvement in discrimination of patients with the use of DBC as compared with original Atlanta classification. DBC offers a >2 times improved stratification of patients with AP, especially those who at highest risk of mortality. Given the arguments mentioned above, this study is well positioned to offer a more comprehensive evidence on the clinical validity of DBC in general and “critical” category in particular.

Our study has some limitations and they need to be acknowledged. First, this pilot study was limited to only to 4 predictors and it is possible that other predictors such as SIRS, BISAP, and BUN may yield a better accuracy in predicting CAP, although a recent systematic literature review showed that their potential to predict persistent OF early in the course of AP is rather limited.^39^ Second, as the study period spanned 4 years, it is unavoidable that the management strategy gradually evolved over the time, especially with regard to the use of percutaneous/endoscopic drainage as opposed to necrosectomy. Third, we only used the value of all parameters during the first 24 hours after hospital admission; hence, the value of continuous measurements of the same parameter or sequential use of different parameters is unknown. Moreover, as APACHE II score is a multifactor system itself and it could not be obtained on admission (as for CRP, IAP, and D-dimer), we did not study the combinations of APACHE II score with the other 3 predictors, which may further improve the performance. Fourth, a considerable part of the included patients were transferred from other hospitals, resulting in a notably high percentage of patients with CAP, which may bring in a selection bias to the study. That is why the main metric that we interpreted was PLR as it is independent of disease prevalence, making it possible to generalize our findings to settings with a low percentage of patients with CAP. Last, some of the included patients had symptoms of AP for up to 96 hours prior to study inclusion; hence, our findings need to be confirmed in other settings. But it is worth noting that the up to 96 hours duration of symptoms has been used often in recent randomized trials of early interventions in AP.^40–42^

In conclusion, APACHE II score ≥11 is the best predictor of CAP within 24 hours of hospital admission. IAP is the most reliable sole predictor that offers a reasonable accuracy. From the perspective of the routine clinical practice, the combination of IAP and CRP appears to be the optimal choice to predict the critical course of AP at the moment. Future studies will need to investigate the value of other novel markers in predicting CAP.
